# Dancing to Metallica and Dora: Case Study of a 19-Month-Old

**DOI:** 10.3389/fpsyg.2019.01073

**Published:** 2019-05-15

**Authors:** Laura K. Cirelli, Sandra E. Trehub

**Affiliations:** ^1^Department of Psychology, University of Toronto Scarborough, Toronto, ON, Canada; ^2^Department of Psychology, University of Toronto Mississauga, Mississauga, ON, Canada

**Keywords:** music, infancy, rhythm, movement, dance, development

## Abstract

Rhythmic movement to music, whether deliberate (e.g., dancing) or inadvertent (e.g., foot-tapping), is ubiquitous. Although parents commonly report that infants move rhythmically to music, especially to familiar music in familiar environments, there has been little systematic study of this behavior. As a preliminary exploration of infants' movement to music in their home environment, we studied V, an infant who began moving rhythmically to music at 6 months of age. Our primary goal was to generate testable hypotheses about movement to music in infancy. Across nine sessions, beginning when V was almost 19 months of age and ending 8 weeks later, she was video-recorded by her mother during the presentation of 60-s excerpts from two familiar and two unfamiliar songs presented at three tempos—the original song tempo as well as faster and slower versions. V exhibited a number of repeated dance movements such as head-bobbing, arm-pumping, torso twists, and bouncing. She danced most to Metallica's *Now that We're Dead*, a recording that her father played daily in V's presence, often dancing with her while it played. Its high pulse clarity, in conjunction with familiarity, may have increased V's propensity to dance, as reflected in lesser dancing to familiar music with low pulse clarity and to unfamiliar music with high pulse clarity. V moved faster to faster music but only for unfamiliar music, perhaps because arousal drove her movement to familiar music. Her movement to music was positively correlated with smiling, highlighting the pleasurable nature of the experience. Rhythmic movement to music may have enhanced her pleasure, and the joy of listening may have promoted her movement. On the basis of behavior observed in this case study, we propose a scaled-up study to obtain definitive evidence about the effects of song familiarity and specific musical features on infant rhythmic movement, the developmental trajectory of dance skills, and the typical range of variation in such skills.

## Introduction

Active musical engagement, whether by attentive listening, singing, or rhythmic movement, is pervasive across the lifespan and across cultures (Savage et al., 2015; Trehub et al., 2019). These activities play a critical role in mood regulation (Laukka, 2007), social bonding (Cirelli et al., 2014; Tarr et al., 2014), personal identity (North and Hargreaves, 1999), and other aspects of well-being (Groarke and Hogan, 2016). The seeds of such musical engagement are planted in infancy when the primary caregiver serves as musical partner and mentor (Cirelli and Trehub, 2018; Trehub and Gudmundsdottir, 2019). Caregivers around the world sing to infants in a warm and distinctive manner (Trehub and Trainor, 1998; Ilari, 2005) that is often accompanied by rhythmic movement (Longhi, 2009). Infants are highly engaged by the infant-directed singing style (Trainor, 1996; Costa-Giomi, 2014) and by the songs themselves. Unfamiliar individuals who sing one of the caregiver's songs elicit more social interest (Mehr et al., 2016) and assistance (Cirelli and Trehub, 2018) from infants than those who sing an unfamiliar song. Although infants exhibit more rhythmic movement to music than to speech (Zentner and Eerola, 2010), the factors that underlie early movement to music remain poorly understood. The present case study was motivated by an interest in generating testable hypotheses about the development of movement to music in infancy.

Moving to the beat of music requires precise temporal processing and auditory-motor integration, including predictive timing and planning at various levels of the motor system (Patel and Iversen, 2014). Music listening, even without movement, activates motor areas in the adult brain (Grahn and Brett, 2007; Fujioka et al., 2009). The newborn's brain tracks sound onsets, offsets, and musical tempo (Winkler et al., 2009; Háden et al., 2015). By 2 months, infants differentiate rhythmic patterns (Demany et al., 1977) and tempo differences (Baruch and Drake, 1997) and, by 12 months, culture-specific exposure influences rhythm perception (Hannon and Trehub, 2005).

Infants exhibit spontaneous movement to metrically regular sounds by 5 months of age, but their movements are not aligned with the beat and change little between 5 and 24 months of age (Zentner and Eerola, 2010). Although infants' pace of movement is reportedly faster for faster music than for slower music (Zentner and Eerola, 2010), that does not seem to be the case for preschoolers (Eerola et al., 2006). In general, mature synchronization to music is typically achieved by 10 years of age (Drake et al., 2000; McAuley et al., 2006). Children's preferred tempo is faster at younger than at older ages, and the tempo range over which they can coordinate their attention and movement increases progressively with age (Drake et al., 2000; McAuley et al., 2006). Little is known, however, about infants' preferred tempo.

*Groove* is often used to describe musical patterns that evoke a desire to move (Madison, 2006). Music rated high in groove activates motor areas in the brain (Stupacher et al., 2013), prompting spontaneous bursts of coordinated movement (Janata et al., 2012). High-groove music tends to have moderate rhythmic complexity (Witek et al., 2014), tempo corresponding to spontaneous tapping rates (Madison et al., 2011; Etani et al., 2018), high pulse clarity, and predominant energy in low-frequency bands (Burger et al., 2013; Stupacher et al., 2016). It is unclear, however, whether those musical features promote rhythmic movement in infants and young children.

If musical tempo influences infants' movement tempo (Zentner and Eerola, 2010), those links may be mediated by arousal (i.e., excitement) and pleasure. Music listening activates adult brain networks linked to pleasure and reward (Blood and Zatorre, 2001). Moreover, dancing to music increases pleasure relative to music listening alone (Dunbar et al., 2012; Bernardi et al., 2017). Musical engagement clearly evokes pleasure in infants. By 5 months, infants smile considerably more to interactive singing than to speech (Mehr et al., 2016). Infants also smile more when moving to music, especially during periods of greater coordination to the music (Zentner and Eerola, 2010).

Gaps in knowledge about infants' movement responses to music, such as the role of music familiarity, testing context, arousal, and pleasure should be addressed systematically. For the moment, however, there is a disconnect between the available laboratory research, which reveals relatively limited movement to music and no apparent change between 5 and 24 months (Zentner and Eerola, 2010), and parent reports of frequent infant “dancing” to music along with dramatic change over the first 2 years. A similar disconnect was evident between young children's reported inability to match the pitch contours and pitch range of songs (e.g., Rutkowski and Miller, 2002) and toddlers' effective matching of those features when singing their favorite songs at home (Gudmundsdottir and Trehub, 2018). As a preliminary exploration of infants' movement to music in their home environment, we undertook a case study of V, a Canadian girl who reportedly began moving rhythmically to music at 6 months of age and joined our study when she was almost 19 months of age. Across nine sessions, V's mother video-recorded her during the presentation of musical excerpts of familiar and unfamiliar songs at three tempos—the original song tempo as well as a faster and slower tempo. Our immediate goal focused on the influence of song familiarity, song type, and tempo on V's duration and tempo of movement as well as possible links between movement to music and visible pleasure. Our broader goal was to generate hypotheses for further research in home or laboratory environments.

## Methods

### Participant

V is part of a bilingual (French/English) household in a small city in Northern Ontario, where she lives with her mother, father, and 7-year-old brother. She was 18 months, 27 days of age on the first recording session and 20 months, 18 days on the final session. According to maternal report, she began moving regularly to music at 6 months of age. V had not participated in music programs in the community, but her parents and brother exposed her to music by singing and dancing with her and playing musical recordings. No member of her family had formal music training, but her father is a self-taught amateur guitarist. Written informed consent was obtained from the mother for the publication of this case report and the inclusion of identifiable information including images, age, and gender.

### Stimuli

The stimuli featured four songs, two familiar and two unfamiliar. When asked to report V's “favorite songs”, her parents identified *Dora the Explorer Theme Song* (119 beats per minute [bpm]) (Sitron and Straus, 2010), heard regularly from 6 months (once weekly at study onset, more frequently in earlier months), and Metallica's *Now that We're Dead* (128 bpm) (Hetfield and Ulrich, 2017), heard regularly from 12 months (once daily at study onset), resulting in greater cumulative exposure to Dora but more consistent recent exposure to Metallica. The *Dora* theme was typically played from YouTube, but V was visibly excited by the music and relatively disinterested in the visuals. Exposure to *Now that We're Dead* (Metallica), her father's favorite song, was from an audio-recording, but father and daughter often danced together to the song, with father bobbing his head to the music. An unfamiliar song chosen to match *Dora* was *Hey It's Franklin*, the theme song from *Franklin the Turtle*, another animated children's TV show (Cockburn, 2000). An unfamiliar song chosen to match *Metallica* was the Backstreet Boys' *Everybody* (Pop and Martin, 1997), a song for adults that has been used previously to investigate cross-species dancing (Patel et al., 2009). We used Audacity 2.1.0, to create versions of each song at 100 bpm (designated *slow*) and 140 bpm (designated *fast*), maintaining original pitch levels. We equated maximum amplitude across clips, which were comparable in subjective loudness. For *intermediate* tempo, familiar songs were maintained at original bpm, and unfamiliar songs were set to 120 bpm. The usual tempo range for dance music is 94–176 bpm, with 120–150 being most common (van Noorden and Moelants, 1999). Pulse clarity (i.e., strength of rhythmic periodicities) and mean fundamental frequency (F0) were calculated at the intermediate tempo using MIRtoolbox 1.7 running on Matlab 2016a. Metallica and Backstreet had high pulse clarity (0.79; 0.67) and low mean F0 (120 Hz; 203 Hz), respectively. Dora and Franklin had low pulse clarity (0.49; 0.31) and high or low F0 (304 Hz; 225 Hz), respectively. The 12 recordings (4 songs X 3 tempos) were trimmed to 60 s, with 1-s fade-out. The first minute of each recording was always used to optimize familiarity for Metallica and Dora. Except for Metallica, which was instrumental^1^, the other songs contained vocals and instrumentals. Excerpts of these clips at each tempo can be accessed in the supplementary section (see Supplementary Audios 1–4). Individual audio files for each test session had six song clips (trials) separated by 10-s silence. The song clips were ordered across sessions so that each session included two clips at each of the three tempos, and each of the 4 songs as heard at least once but not more than twice. Across all 8 sessions, the 12 clips (4 songs at 3 tempos) were presented 4 times each^2^. A ninth session replaced trials lost because of V's fussiness (four trials) or camera malfunction (one trial).

### Procedure

Audio files for each session were provided via Google Drive. The mother was instructed to play the audio files for V at her convenience and to capture V's responses with video-recordings. She was asked to refrain from moving to the music or singing during the recording sessions and to ensure that no other family members (brother or father, if present) did so. However, she was free to offer verbal encouragement (e.g., asking V to dance). In fact, she provided such encouragement only when V had not initiated dancing on a trial, doing so comparably on familiar (65%) and unfamiliar (65%) song trials. The music was presented (at ~60 dB) by means of a personal tablet, and sessions were recorded by cell phone. After each session, the mother reported time of day, those present, and any notable circumstances, transmitting all recordings and information via Google Drive.

### Data Analysis

**Figure 1:**
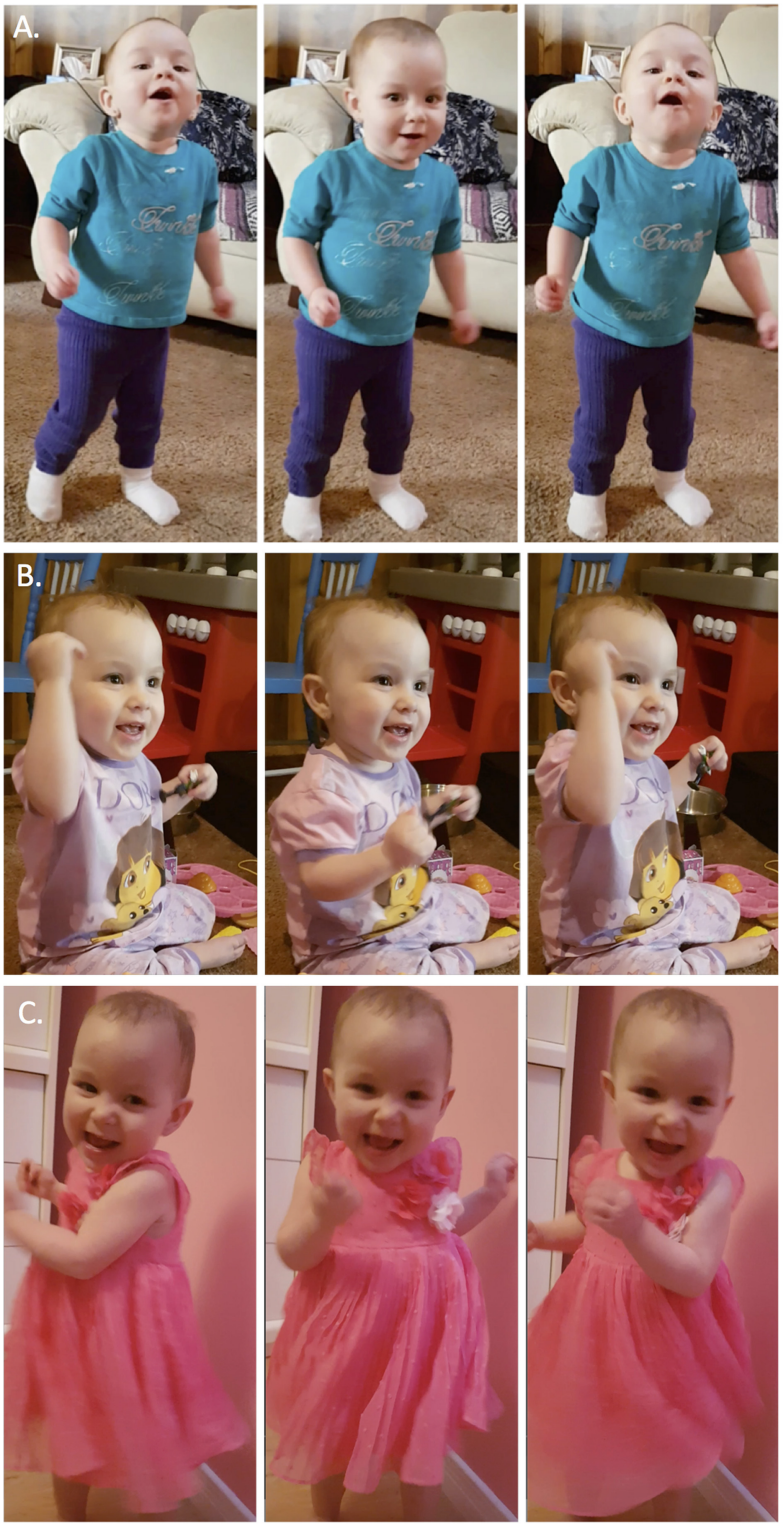
Examples of V's common dance moves. **(A)** Head bobs, **(B)** Arm pumps, and **(C)** Torso twists. Video examples can be found in the Supplementary Materials. Written informed consent obtained from the parents of the child for the publication of these images.

An assistant coded the videos with ELAN software (https://tla.mpi.nl/tools/tla-tools/elan/, Lausberg and Sloetjes, 2009). She was trained extensively by the lead author, and had years of experience working with young children. The assistant used audio only to locate the start of each trial, subsequently coding the target behaviors with the audio turned off. For each trial, she identified number of dance bursts, burst duration, and percentage of time smiling when V's face was visible. Dance bursts were defined as time windows with two or more rhythmic movements within 2,000 ms. Independent coding of 27% of the trials yielded high interrater reliability for the three measures (*r* = 0.97, *r* = 0.98, *r* = 0.82, for number of dance bursts, burst duration, and smiling, respectively). V exhibited various dance patterns including head bobbing, arm pumps, swaying, torso twists, and bouncing (examples in Figure 1). For each dance burst, the coder indicated the time of peak movement position (lowest head position for head-bobbing, fully extended arm for arm pump). V's requests for more music (“d'autre?”) were coded during the silent interval between songs.

## Results

**Figure 2:**
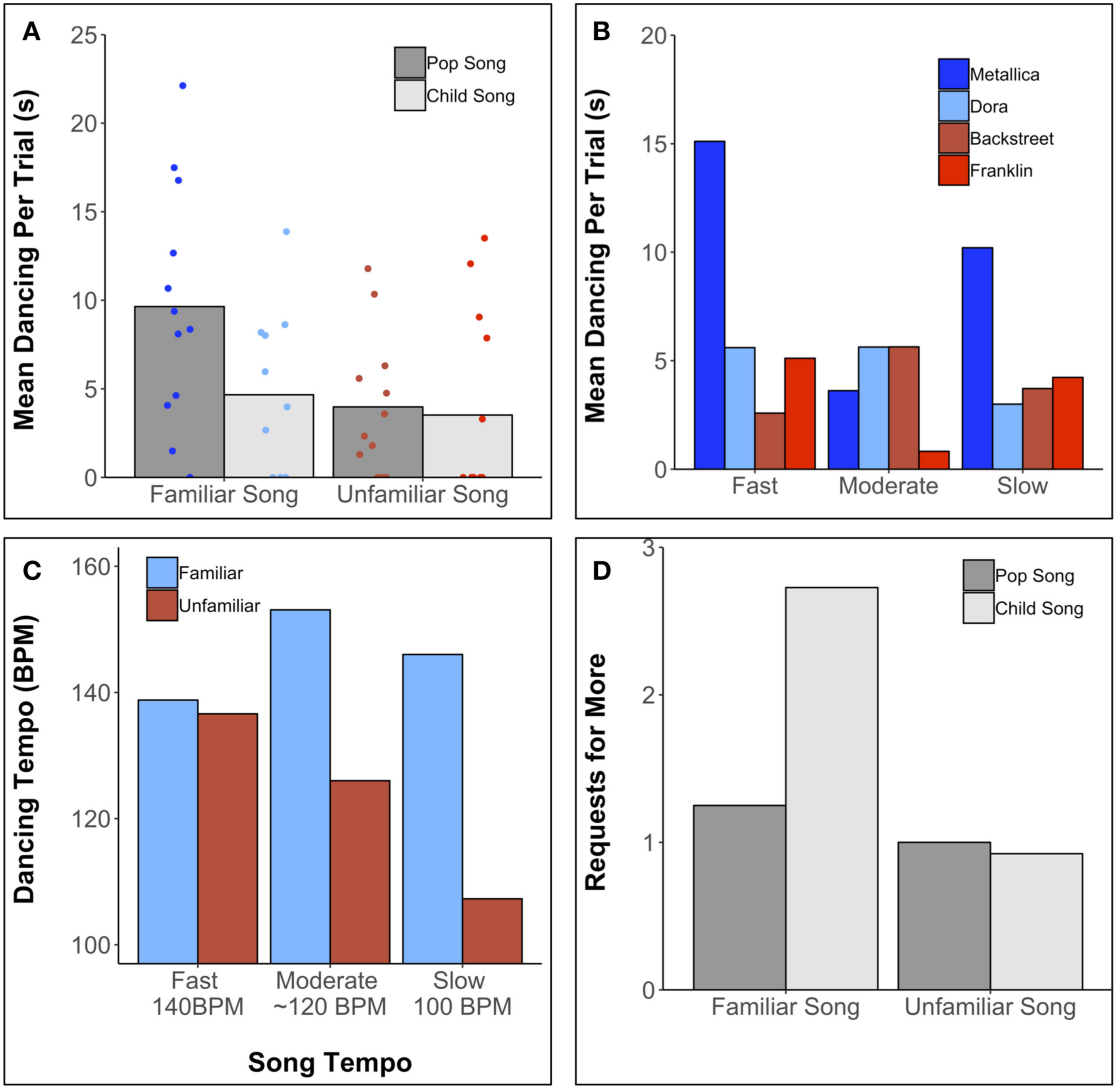
**(A)** Mean time dancing during the 60-s trials for familiar (Metallica, Dora) and unfamiliar (Backstreet Boys, Franklin) songs. Individual trial data are displayed as jittered scatter points. **(B)** Mean time dancing per trial as a function of tempo and song. **(C)** Mean dancing tempo across Song Tempo and Song Familiarity conditions. **(D)** Mean number of requests for more (“d'autre?”) after song trials featuring Metallica (familiar pop song), Dora (familiar children's song), Backstreet Boys (unfamiliar pop song), and Franklin (unfamiliar children's song).

Dance bursts occurred in all recording sessions and in 32 of 48 trials. For trials with dance bursts, bursts per trial ranged from 1 to 5 (*M* = 2.22, *SD* = 1.21). Although dance bursts occurred in 67% of the trials, overall dancing per trial was limited, from 1.3 s to 22.1 s (*M* = 8.1 s, *SD* = 5.1 s for trials with at least 1 dance burst). Mean dancing per trial also varied across song selections. As can be seen in Figure 2A, V danced more to Metallica's *Now That We're Dead*, a familiar pop song (heavy metal genre) that had considerably greater pulse clarity than the other familiar song and lower mean F0 than the other songs.

Dance time as a function of tempo is shown in Figure 2B. V's propensity to dance most to Metallica was driven by the tempo-modified (fast and slow) conditions. Otherwise, tempo exerted no clear effects on dance time.

Table 1 shows the incidence of each recurring dance pattern (see Figure 1 and Supplementary Videos 1–3 for examples) for each song. Arm pumps were common during three of the four songs, and distinctive head-bobbing was especially notable during Metallica trials.

**Table 1:** Number of trials in which V performed specific dance patterns.

**Song**	**Head bobbing**	**Arm pumps**	**Swaying**	**Torso twists**	**Bouncing**
Dora	2	5	0	2	1
Metallica	11	4	0	1	3
Backstreet	3	5	0	3	3
Franklin	1	1	2	2	0

**Figure 3:**
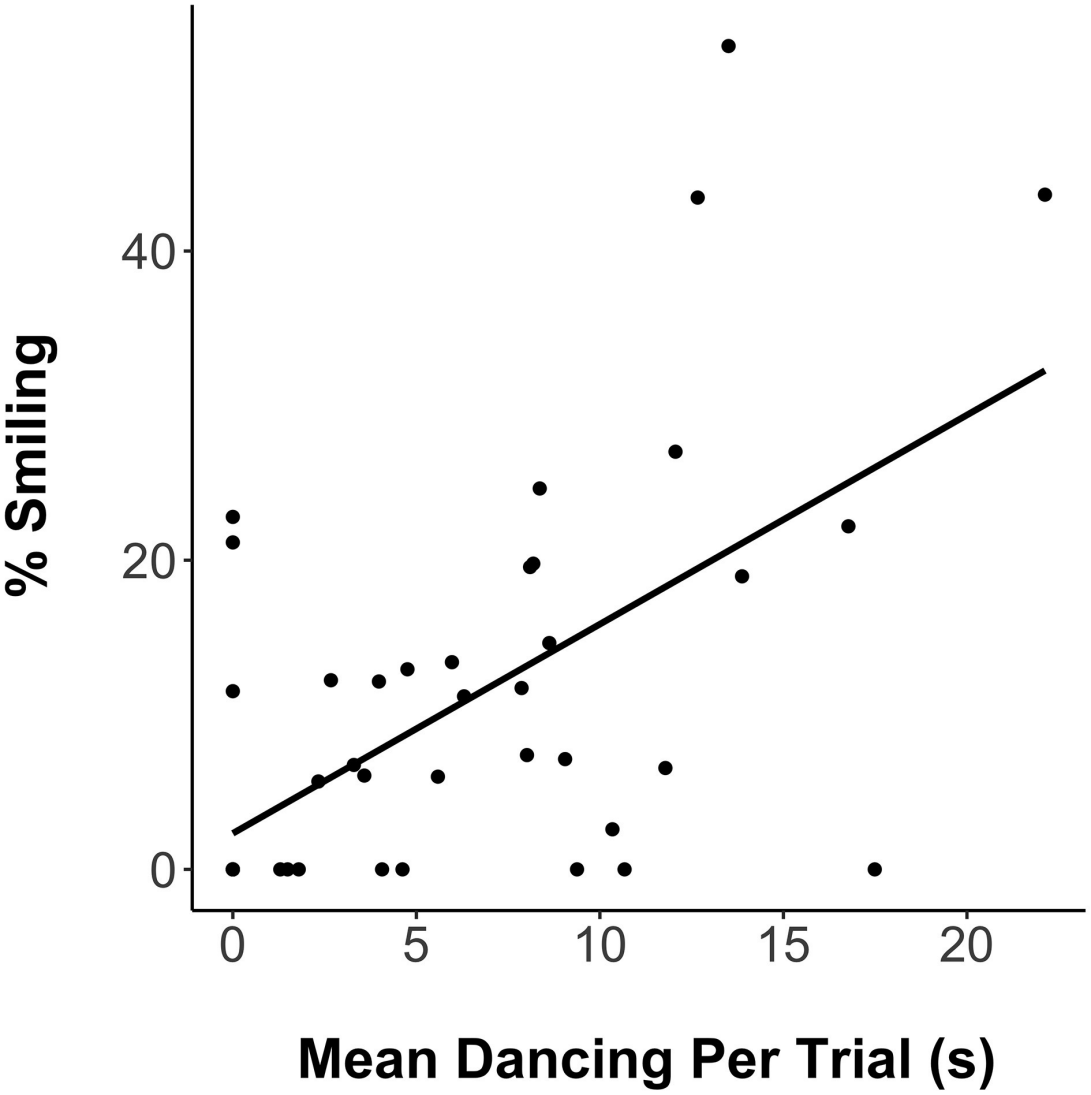
Correlation between dance time per trial (s) and the percentage of time smiling (based on total face-visible time per trial).

A Pearson correlation between dance time and percentage of time smiling per trial, *r* = 0.60, *p* < 0.001 (Figure 3) highlighted the link between musical movement and positive affect.

For each dance burst, we calculated interonset intervals (ms) between successive movement peaks. For each trial, mean interonset interval was converted to movement rate (bpm). Movement rate across tempo (Fast, Moderate, Slow) and song familiarity is displayed in Figure 2C. For familiar songs, movement tempo was rapid for all musical tempos. For unfamiliar songs, there is suggestive evidence of slower movement tempo for slower songs. Moreover, there is a suggestion of tempo convergence (coordination of movement with musical tempo) at the fastest song tempo regardless of familiarity.

During the 10-s silent intervals between songs, V asked for “more” (“d'autre?”) after 27 of 48 trials (56%), doing so more frequently (sometimes repeatedly) after hearing familiar songs (70%) than unfamiliar songs (44%) (see Figure 2D) and most frequently after hearing the Dora song.

## Discussion

The present case study explored the feasibility of studying infant dance or rhythmic movement to music in the home environment. We focused specifically on the influence of song familiarity and tempo on the incidence and tempo of movement as well as links between movement and pleasure. V danced in every recording session, supporting the home environment as a promising venue for eliciting movement to music. Overall, she danced 9% of the time, which is modest but somewhat higher than the 6% of rhythmic movement for 5- to 24-month-olds in a laboratory context (Zentner and Eerola, 2010). Note that the laboratory study excluded data from a third of infants who fussed or attended excessively to the caregiver. Moreover, infants sat on their parents' lap, reducing the likelihood of observing typical patterns of movement to music. As for 2- to 4-year-olds, 40% were unwilling to dance in the laboratory (Eerola et al., 2006).

V's head-bobbing, arm pumping, and body twists were more dance-specific and motorically complex than the rhythmic limb movements observed in previous studies with infants (Zentner and Eerola, 2010; Fujii et al., 2014) but less complex than the rhythmic body movements observed with 2- to 4-year-olds (Eerola et al., 2006). Age-related changes in the complexity of V's “dancing” can be seen in her limb movements at 8 months (Supplementary Video 4) before data collection began, body twists at 19 months (Supplementary Video 5), and hopping at 29 months (Supplementary Video 6) after data collection ended. V's most common dance move was head-bobbing, especially for Metallica. Such motion has powerful effects on the vestibular system and on rhythm perception (Phillips-Silver and Trainor, 2008). Adult head-bobbing is often evoked by music with a strong bass line (Burger et al., 2013), as in the Metallica song. The contribution of bass sounds to the nature and frequency of young children's movements can be explored in future investigations with a wider range of stimuli and a large sample of children across a broad age range. V's head-bobbing to Metallica probably stemmed from dancing with her father, who modeled such head movements to that song.

V's dance duration was greater for familiar music (12% of the time) than for unfamiliar music (6%), which was driven by her response to Metallica (16%). For example, V danced no more to the familiar and well-liked Dora theme than to the unfamiliar excerpts. Familiarity may influence rhythmic movement in infants but only in conjunction with other factors such as pulse clarity or beat salience, which is correlated with groove (Madison et al., 2011; Witek et al., 2014; Stupacher et al., 2016). *Now that We're Dead* has high pulse clarity, in contrast to the low pulse clarity of Dora. On its own, high pulse clarity may be insufficient to enhance movement in infants, as evident in V's modest dance duration to *Everybody* (Backstreet Boys), which was high in pulse clarity but unfamiliar. V's experience with *Now that We're Dead* as a “dance song” may be relevant. The relative contributions of familiarity and pitch clarity to movement could be explored in future research that involves independent manipulation of these factors.

Unexpectedly, V danced most to Metallica on trials with altered tempo (21%)−15.1 s with increased tempo, 10.2 s with decreased tempo, and 3.6 s with the original tempo. Presumably she had long-term memory for the original tempo, perhaps finding the altered versions amusing or exciting, as reflected in increased smiling during the altered versions. Just as younger infants perceive absurd versions of familiar events as humorous (Mireault et al., 2018), older infants may react with heightened excitement when their expectations of highly familiar music are violated. Musical tempo may be most memorable for music with a highly salient bass line or pulse and least memorable for music with a less salient bass line and more salient melody line.

With respect to tempo flexibility, V's tempo of movement was faster for the familiar songs than for the unfamiliar songs (see Figure 2C), perhaps because of heightened arousal. For the familiar songs, moreover, V danced fastest for the original tempo and slowest for the fastest tempo, precluding orderly relations between dance tempo and musical tempo. For the unfamiliar music, there was tentative evidence of tempo flexibility in the sense of faster dancing to faster music. Infants may accord greater attention to timing details in the context of unfamiliar songs that elicit moderate arousal levels. Although 5- to 24-month-olds move modestly faster to faster music than to slower music (Zentner and Eerola, 2010), preschool children show little evidence of doing so (Eerola et al., 2006). Further study of the effects of age, song familiarity, and social context (dancing alone or with parent) on tempo flexibility is warranted.

Developmental changes in coordinated movement to music have been linked to changes in spontaneous tempo (characteristic rate of rhythmic movement without sound) and preferred tempo (optimal tempo for perception and coordinated movement) (McAuley et al., 2006). V's movement timing was closest to the target tempo for the fastest songs (140 bpm), both familiar and unfamiliar (Figure 2C). Spontaneous motor tempo changes from ~200 bpm at 4 years of age to 90 bpm in elderly adults, and synchronization is most accurate when the target tempo matches the preferred tempo (McAuley et al., 2006). The developmental timetable may be influenced by age-related changes in internal timing mechanisms (Vanneste et al., 2001; McAuley et al., 2006) and biomechanical factors involving limb length and weight (Goodman et al., 2000). Future research with infants should encompass a wider range of tempos, including the preferred tempo of 4-year-olds (200 bpm), and spontaneous motor tempo should be documented.

V's dance duration per trial was positively related to smiling (Figure 3), highlighting the links between dance and pleasure. In previous research, smiling was related to ratings of movement coordination to music in 5- to 24-month-olds (Zentner and Eerola, 2010). Does movement to music generate pleasure, or do pleasurable responses to music motivate movement? Both may well be the case. Adults' motivation to move to music has been linked to pleasure and to perceived musical groove (Janata et al., 2012; Witek et al., 2014). Musical features such as syncopation have independent effects on pleasure and desire to move (Witek et al., 2014). Moreover, musical movement heightens pleasure through increased arousal (Tarr et al., 2015).

Although Metallica prompted V to dance more and to dance distinctively, Dora prompted the most requests for more music. Aside from its familiarity, the high pitch and prominent vocals may have contributed to its appeal. Infants' preference for high pitch in speech (Fernald, 1992) and song (Trainor and Zacharias, 1998) is well-documented.

Obviously, a case study of a normally developing child warrants cautious interpretation and cannot speak to issues of individual differences or to development in general. Nevertheless, V's responses reveal dance behavior that is possible but not necessarily typical for 19- or 20-month-olds. A study of supine 3- and 4-month-olds revealed less movement in the context of music than in silence, but 2 of the 30 infants moved more to the music and also exhibited some coordination to the musical beat (Fujii et al., 2014). That study underlines the importance of considering individual behavior patterns even in group studies. With respect to the present study, a scaled-up version with large sample size could provide definitive evidence about the effects of song familiarity and various musical features on infant movement. When familiarity and pulse clarity are manipulated independently, we would expect familiar music to prompt more dancing than unfamiliar music and music with high pulse clarity to prompt more dancing than music with low pulse clarity. In light of the current findings, we would also expect young children to demonstrate greater tempo flexibility when moving to unfamiliar compared to familiar music. An expanded age range (e.g., 1–4 years of age) could provide information about the developmental trajectory of dance skills (e.g., characteristic movements, tempo flexibility, synchrony), the typical range of variation, and the effects of dance experience and musical exposure. Systematic testing in the home environment with specified, parent-administered protocols would provide multiple benefits such as a comfortable and familiar context for participating children, convenience for parents, access to non-local as well as local participants, and a wealth of information about the early development of dancing.

## Ethics Statement

This study was carried out in accordance with the recommendations of the University of Toronto Research Ethics Board with written informed consent from the participant's legal guardian.

## Author Contributions

Both LC and ST contributed to study design, data analysis, and manuscript preparation.

### Conflict of Interest Statement

The authors declare that the research was conducted in the absence of any commercial or financial relationships that could be construed as a potential conflict of interest.

## Footnotes

^1^The first minute of the original recording is instrumental.

^2^An error resulted in having 3 Dora Fast trials and 5 Franklin Fast trials.

## Supplementary Material

Supplementary Video 1 The arm pump.

Supplementary Video 2 The head bob.

Supplementary Video 3 The torso twist.

Supplementary Video 4 V at 8 months responding to *Dora*.

Supplementary Video 5 V at 19 months responding to *Dora*.

Supplementary Video 6 V at 29 months responding to *Dora*.

Supplementary Audio 1 30-s clips of *Dora* at slow, moderate, and fast tempos.

Supplementary Audio 2 30-s clips of *Metallica* at slow, moderate, and fast tempos.

Supplementary Audio 3 30-s clips of *Franklin* at slow, moderate, and fast tempos.

Supplementary Audio 4 30-s clips of *Everybody* at slow, moderate, and fast tempos.
